# Cases in Ophthalmic Practice

**Published:** 1862-06

**Authors:** E. L. Holmes

**Affiliations:** Chicago


### ORIGINAL COMMUNICATIONS.

### CASES IN OPHTHALMIC PRACTICE.

By E. L. HOLMES, M. D., of Chicago.

*Occlusion of Pupil and Cataract.*—A young man about twenty four years of age was severely burned in the face and eyes, some months since, by the premature discharge of a cannon. He was totally blind for several weeks. After the injuries had healed, four months after the accident, he came under my care. I found the cornea of the left eye so completely covered with opacities that the iris and pupil were scarcely discernible. Yet, at the upper part of the cornea, there was a small transparent portion; from the fact that the patient could perceive but little light through this part of the cornea, there was reason to suspect that the shock and inflammation had seriously injured the retina, There was really no hope that any plan of treatment for this eye could restore vision.

The other cornea, however, was perfectly transparent, with the exception of a few minute opaque spots on its outer half, produced, without doubt, by grains of powder. The iris had evidently suffered from inflammation, since it was somewhat discolored, the pupil being much contracted, irregular, and attached to the lens, which was quite opaque. Atropine had scarcely any effect in dilating the pupil. With this eye the patient could barely see to walk with the assistance of a staff, where he was acquainted. The palpebral conjunctivæ of each eye were slightly granulated; but the sulphate of copper, applied daily in crystal, soon restored the lid to a healthy condition.

The double operation, for artificial pupil and for cataract, alone was apparently indicated. The first operation was performed by making a small incision, less than a sixth of an inch in length, into the anterior chamber through the nasal side of the sclerotic, about a sixteenth of an inch behind its union with the cornea. By means of a small-toothed forceps, introduced into the anterior chamber, a piece of the iris was torn away and excised, leaving quite a well formed pupil situated at the inner side of the iris. No inflammation followed, and in a few days the patient was able to walk out with the eye shaded. At the expiration of a month the cataract was thoroughly broken up by a needle passed through the sclerotic, and in a short time absorption had so far progressed, that the pupil became quite clear and vision much improved. In a few weeks more the patient was able, with the assistance of a cataract glass of five inch focus, to see distant objects distinctly, and read or write with a glass of two and a half inch focus.

*Operation for Soft Cataract—iris and lens adherent.*—F. H. a carpenter from the country, German, aged about 25 years, had suffered from cataract for more than a year. In December last he had been unable for three months to discern objects with this eye. The other eye had also become quite dull from the same condition of the lens. I found the left pupil active under the influence of light, but irregular; the lens very opaque, of a uniform milky color, and not much enlarged, as is often the case in cataracts which form rapidly. The iris was attached to the nasal side of the lens, nearly a sixth of an inch in extent. The patient had never experienced pain in or around the eye, and was not aware that the iris had ever been inflamed. I determined to break up the cataract freely by means of a needle passed through the sclerotic. As adhesions between the iris and lens are sometimes quite readily broken, without injury to the iris, I hoped to be able to separate the cataract from the iris with the needle. Unfortunately the capsule of the lens resisted the needle more than I anticipated. In breaking up the lens the iris was put considerably upon the stretch, and, I feared, somewhat injured. The lens, however, was completely broken up and fragments projected through the pupil. The central portions of the cataract seemed reduced almost to a fluid condition. The next day the eye was somewhat congested; the pupil was contracted and the patient complained of considerable pain in the eye. As excision of a portion of the iris (iridectomy) has been shown to render the iris less liable to inflammation after injury, I at once resolved to excise a portion of this membrane and to extract the fragments of the lens. After administering chloroform, I made a straight incision about a quarter of an inch in length through the nasal side of the cornea as near as possible to the sclerotic, and as in the preceding case, removed a portion of the iris. By means of a small "scoop" the entire cataract was removed, leaving the pupil perfectly clear. The next day the pain had entirely subsided. The patient was kept in a darkened room for three weeks, when the incision had healed, and vision, even without a convex lens, was found to be remarkably good. The pupil, of course, was somewhat irregular. In three months the patient returned to the city for me to select a suitable lens. With one of three and a half inch focus, he was able to pursue his ordinary occupation.

*Pannus, granulations, &c., of eight years standing.*—The following case is reported simply as an example of the recuperative powers of the system in certain diseases of the eye. An Irishman, about 47 years of age, was attacked at the East, as he stated, eight years previous to consulting me, with inflammation of the conjunctiva. In spite of all treatment he became blind. He was subsequently treated in New York and other places at the East and also in Chicago without benefit. For eight years he had been unable to do a day's work. His health had suffered seriously from want of exercise and from confinement in the house. At the time I first saw him, both corneæ were so densely covered with vessels as to conceal the pupils; the palpebral conjunctiva of the lids of each eye was not only granulated, but also atrophied; the lower cilia being very irregular, and directed against the globe. The severity and duration of the disease and the ill health of the patient rendered the prognosis as regards the eyes very unfavorable. By removing a portion of the integument immediately below the cilia of each lower lid, the trichiasis was removed. The daily application of the sulphate of copper in crystal to the inverted lids, reduced the granulations and caused absorption of the vessels and organized lymph of the cornea. Especial attention was directed to regular exercise in the open air, good diet and the use of tonics. The general health soon improved; the cornea at the end of three months became quite clear, and in less than a year had resumed its normal transparency. The iris and lens had remained in a healthy condition for eight years during all this protracted disease.

